# Chicago Veterinary College

**Published:** 1897-05

**Authors:** 


### CHICAGO VETERINARY COLLEGE.

On Wednesday, March 24th, the students and the graduating class of 1896–'97 were given a banquet by the faculty at the Sherman House. Justice having been done to the good things set before the participants, speeches and toasts emphasizing the good feeling existing between the faculty and class were indulged in, and at a late hour the banquet hall was vacated, a most delightful evening having been spent. On Thursday, March 25th, the commencement exercises were held in the college auditorium, the large hall being specially decorated for the occasion. The audience, consisting of the friends of the faculty and graduating class, thronged the lecture-room, filling it to its utmost capacity. The class colors —purple and white—were conspicuously worn by the majority of those present. The proceedings opened by instrumental music by the Lawndale quartette. On the platform were grouped all the members of the faculty, the chair being occupied by Dr. Joseph Hughes, who, in his opening remarks, briefly related the history of the school, and highly complimented the graduating class on the splendid showing made by them, of the twenty-two graduates thirteen having passed with honors, of which the following is a list according to their standing: Drs. M. J. Dunleavy, J. H. Oliphant, G. P. Frost, F. W. Benteen, Jr., F. L. Cusack, E. T. Frank, George Fry, James Smellie, C. A. Bradley, V. A. Holden, H. J. Schneider, W. F. Fish, and T. J. Menestrina.

Special congratulations were tendered to Dr. M. J. Dunleavy, who obtained the gold medal for the highest general average; to Dr. J. H. Oliphant, who obtained first prizes in the theory and practice of chemistry; and to Dr. George P. Frost, who obtained the prize in anatomy. Having made this announcement, the chairman conferred the degree, M.D.C. (Doctor of Comparative Medicine), and delivered diplomas to the following members of the graduating class: J. H. Oliphant, Macon, Ga.; William Sonerol, Ludington, Mich.; A. M. Taylor, Chicago, Ill.; L. D. Brown, Hamilton, Mo.; F. L. Cusack, Milwaukee, Wis.; E. T. Frank, Warren, Minn.; T. J. Menestrina, St. Louis, Mo.; C. F. Gruner, Chicago, Ill.; James O'Donnell, Milwaukee, Wis.; C. A. Bradley,

Wyoming, Ia.; W. H. Scruby, St. Cloud, Minn.; James Smellie, Chicago, Ill.; F. W. Benteen, Jr., Atlanta, Ga.; F. A. Ramsey, Encinitas, Cal.; M. J. Dunleavy, Denver, Col.; H. J. Schneider, Milwaukee, Wis.; W. J. Martin, Kankakee, Ill.; L. W. Young, Chicago, Ill.; G. P. Frost, Dublin, Ireland; George Fry, Naperville, Ill.; W. F. Fish, Chicago, Ill.; V. A. Holden, Sparta, Wis.

When the members of the class had resumed their seats, Dr. E. T. Frank gave a reading, "The Horse's Troublous Life," which was well received. Dr. M. H. Trumbower, in well-chosen and humorous remarks, then distributed the prizes, many beautiful floral tributes being sent by friends of the young veterinarians. A vocal duet and piano solo were then followed by the class prophecy, the prophet being Dr. M. J. Dunleavy, who, in an exceedingly clever and witty production, held up the little peculiarities of his various fellow-students and produced much merriment by his predictions. The valedictory address was a master effort, and was delivered by Dr. L. W. Young, who, during its delivery, on many occasions received the plaudits of the audience. Dr. A. S. Alexander, being absent owing to illness, Dr. J. F. Ryan delivered the doctorate address, and was followed by Dr. A. H. Baker, who gave some healthy advice to the young graduates.

